# False-Negative 18F-Fluorodeoxyglucose PET/CT in Malignant Pleural Mesothelioma

**DOI:** 10.7759/cureus.17263

**Published:** 2021-08-17

**Authors:** Haley Corbin, Clifford D Packer

**Affiliations:** 1 Internal Medicine, Case Western Reserve University School of Medicine, Cleveland, USA

**Keywords:** malignant pleural mesothelioma, false-negative, pet/ct, pleural biopsy, pleural effusion

## Abstract

We report a diagnostically challenging case of a 77-year-old man who presented with shortness of breath and was found to have a large right hydropneumothorax with collapse of the right lung. A malignancy was suspected, but pleural fluid cytology and 18F-fluorodeoxyglucose (FDG) positron emission tomography (PET)/CT imaging were negative. He then underwent video-assisted thoracoscopy with biopsies of the pleura and chest wall which revealed malignant pleural mesothelioma (MPM). Older patients with early stage MPM are more likely to have false-negative FDG PET/CT results. Pleural biopsy is essential when there is clinical suspicion for mesothelioma, even with negative initial FDG PET imaging.

## Introduction

We report a diagnostically challenging case of malignant pleural mesothelioma (MPM) with negative pleural fluid cytology and negative 18F-fluorodeoxyglucose (FDG) positron emission tomography (PET)/CT imaging that was finally diagnosed by video-assisted thoracoscopic pleural biopsy. Older patients with early stage MPM are more likely to have false-negative FDG PET/CT results [1]. Pleural biopsy is essential when there is clinical suspicion for mesothelioma, even with negative initial FDG PET imaging.

## Case presentation

**Figure 1:**
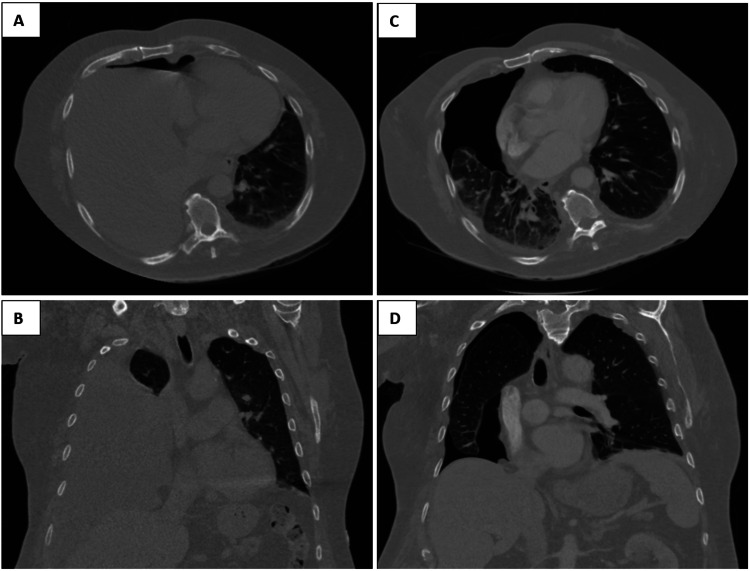
CT imaging before (A and B) and after (C and D) thoracentesis. A and B show the large right hydropneumothorax seen on initial CT imaging. C and D illustrate failure of the right lung to re-expand upon removal of fluid.

**Figure 2:**
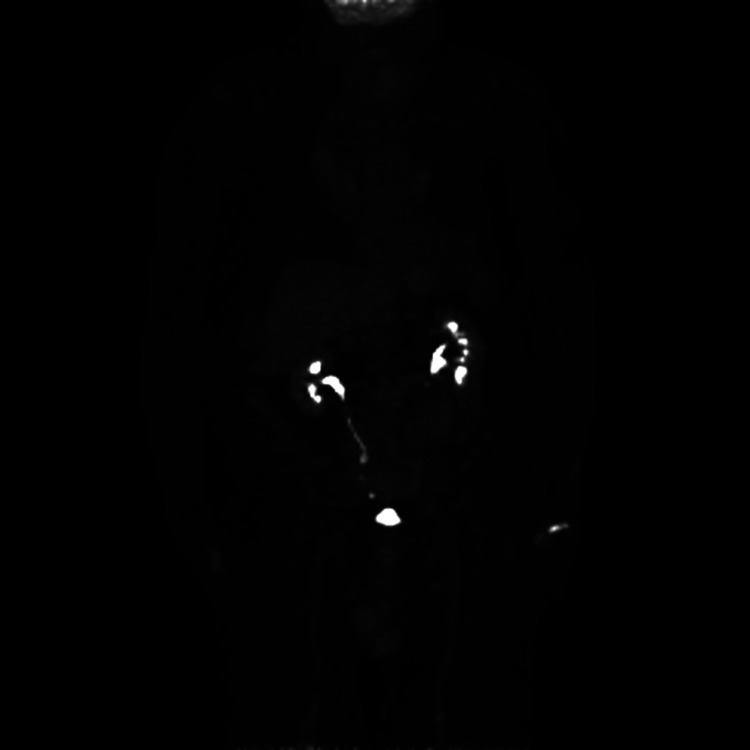
FDG PET/CT image revealing no evidence of malignancy or metastases. FDG: 18F-fluorodeoxyglucose; PET: positron emission tomography

A 77-year-old man with a past medical history of hypertension, hyperlipidemia, psoriasis, and osteoarthritis and an occupational history of welding presented with one month of progressively worsening shortness of breath. On initial evaluation in the Emergency Department (ED), the patient was afebrile and hemodynamically stable with a heart rate of 99, respiratory rate of 22, blood pressure of 149/77, and oxygen saturation of 81% on room air, which improved to 92% when initiated on 3 L O_2_ via nasal cannula. Chest CT revealed a large right hydropneumothorax and collapse of the right lung with a 23 mm subcarinal lymph node and several 2-3 cm hypodensities in the liver. His presentation was concerning for malignant pleural effusion with possible metastatic disease to the liver. Nearly 2 L of fluid was aspirated by thoracentesis, with cytology negative for malignant cells. The pleural fluid protein was 4.4 g/dL, serum protein was 6.1 g/dL, and pleural fluid cholesterol was 138 mg/dL, consistent with an exudate by Light’s criteria. Additionally, the right lung failed to re-expand following removal of the pleural effusion (Figure 1). Workup of the cause of his large hydropneumothorax and trapped lung was unrevealing, including negative rheumatologic markers (rheumatoid factor (RF), anti-cyclic citrullinated peptide (CCP), and anti-nuclear antibody (ANA)), HIV serology, Quantiferon, and Histoplasma urine antigen testing. PET/CT was unremarkable with no convincing evidence of FDG-avid malignancy or metastases (Figure 2). Further, no abnormal metabolism was observed in the enlarged subcarinal node or right hepatic lobe hypodense lesions, which were thought to be benign cysts. Despite negative pleural fluid cytology and PET results, clinical suspicion for mesothelioma was high based on the initial chest CT findings, and the decision was made to proceed with video-assisted thoracoscopic surgery. Biopsies of the right chest wall, parietal pleura, and pleural rind all revealed malignant mesothelioma, epithelioid type. A repeat thoracentesis was again negative for malignant cells, and endobronchial ultrasound revealed no malignant cells on bronchial washing and 3/3 lymph node fine needle aspirations were negative. Despite the negative thoracentesis and endobronchial ultrasound findings and lack of evidence of distant metastases, the tumor was deemed to be surgically unresectable because of encasement and entrapment of the right lung. The patient was discharged with a PleurX catheter (CareFusion, San Diego, CA) and plan to initiate chemotherapy with cisplatin and pemetrexed as an outpatient.

## Discussion

Our case illustrates the challenges of early diagnosis of MPM in patients presenting with a new exudative pleural effusion. Our patient had false-negative results for both pleural fluid cytology and PET/CT imaging, and required video-assisted thoracoscopy with pleural and chest wall biopsies to make the diagnosis. The sensitivity of pleural fluid cytology for MPM is low, ranging from 30% to 51% [2-3]; it is slightly higher in patients with the epithelioid subtype (54%) as opposed to the sarcomatoid subtype (20%) [2]. The difficulties raised by false-negative cytology results are compounded by the potential for false-negative results on PET/CT imaging. A 2015 meta-analysis investigating diagnostic accuracy of FDG PET in differentiating benign from malignant pleural effusions reported 60-100% sensitivity of PET/CT in patients with MPM; however, this analysis was limited by a small number of studies, small sample sizes ranging from 6 to 47 MPM patients, and inconsistent maximum standardized uptake value (SUVmax) cutoff levels [4]. Notably, this study called for future studies to identify the role of PET/CT in “the most challenging clinical scenario” exemplified by this case which lacked clear pleural nodularity or thickening on CT with negative pleural fluid cytology. A more recent study by Lococo et al. (2020) retrospectively evaluated the diagnostic performance of FDG PET in 141 patients diagnosed with MPM over a nine-year period and found an 11.6% false-negative rate. In Lococo et al.’s study, both advanced age and early stage of disease were found to be independent predictors of false-negative PET/CT results. One-third of the patients presenting with stage I MPM had false-negative PET/CT results, and mean age in PET-negative cases was 74 versus 68 in PET-positive cases. Additionally, lower FDG uptake was observed in patients with epithelioid subtype versus biphasic or sarcomatoid subtypes [1]. Our patient displayed a combination of these factors associated with false-negative results as he was 77 years old at diagnosis with epithelioid subtype MPM and T3 tumor with no evidence of metastases or lymph node involvement (stage IB). The underlying reason why age seems to predict PET/CT sensitivity in MPM diagnosis is not entirely clear but may involve inflammatory changes associated with aging which could obscure PET interpretation, as well as lower tumor metabolic activity perhaps influenced by age-related changes in glucose metabolism and specific tumor histology. Small tumor size likely also contributes to false-negative FDG PET/CT results in MPM; our patient had extensive chest wall and pleural involvement, but no masses were seen on imaging. Key factors influencing FDG uptake that may also be at play include differences in levels of glucose membrane transporters for FDG uptake, hexokinase which traps FDG in cells, and glucose-6-phosphatase [5].

Although the use of FDG PET/CT in detecting malignant lung nodules is well-established, its diagnostic utility in MPM is less studied and more difficult to assess large-scale. According to 2018 American Society of Clinical Oncology (ASCO) guidelines, diagnosis of MPM is made using thoracentesis with cytology of fluid or thoracoscopic biopsy, while FDG PET/CT should be obtained as part of initial staging [6]. In addition to its value in staging MPM, SUVmax on FDG PET/CT has been shown to have prognostic value, predicting overall survival [7], especially in MPM of the epithelioid subtype [8].

## Conclusions

In conclusion, PET/CT is a useful tool in staging and prognosis of MPM and may aid in MPM diagnosis. However, reliance on pleural fluid cytology and PET/CT results alone may delay the diagnosis of MPM in patients with suspicious pleural effusions. This could lead to a delay in treatment for patients with early disease and potential for a more favorable response to treatment. This case illustrates the importance of obtaining a biopsy despite negative pleural fluid cytology and PET/CT results when clinical suspicion for mesothelioma remains, especially in older patients with early stage disease.
